# Which has a Greater Influence on Smile Esthetics Perception: Teeth or Lips?

**Published:** 2013-10

**Authors:** Fahimeh Farzanegan, Arezoo Jahanbin, Hadi Darvishpour, Soheil Salari

**Affiliations:** 1 Dental Material Research Center, Faculty of Dentistry, Mashhad University of Medical Sciences, Mashhad, Iran.; 2 Dental Research Center, Mashhad University of Medical Sciences, Mashhad, Iran.; 3 Orthodontist, Tehran, Iran.

**Keywords:** Orthodontics, Smile, Esthetics

## Abstract

**Introduction:**

The aim of this study was to evaluate the role of teeth and lips in the perception of smile esthetics.

**Materials and Methods:**

Thirty women, ranging between 20 and 30 years of age, all with Class I canine and molar relationships and no history of orthodontic treatment, were chosen. Five black and white photographs were taken of each participant in a natural head position while smiling. The most natural photo, demonstrating a social smile, was selected. Two other photographs were also taken from a dental frontal view of each subject using a retractor, as well as a lip-together smile. Three groups of judges including 20 orthodontists, 20 restorative specialists, and 20 laypersons were selected. The judges were then asked to confirm the esthetics of each picture on a visual analogue scale. An analysis of variance (ANOVA) and the Pearson correlation test were used for statistical analysis.

**Results:**

For the orthodontists group, correlation between the scores given to the full smile and each of its components was significant (α=0.05), with equal correlation of each component with the full smile. In contrast to laypersons, the correlation between the scores given to the full smile and each of its components among restorative specialists was significant.

**Conclusion:**

For orthodontists and restorative specialists, esthetic details and the components of the smile (teeth and perioral soft tissues) were important in esthetics perception. In contrast, laypersons perceived no effect of esthetics detail or smile components.

## Introduction

There are numerous factors contributing to smile esthetics. In fact, the upper and lower lips make a frame around the teeth, gingiva, and the oral cavity to form the overall smile parameters. In previous studies, the effects of lip shape (1), smile style (2,3), smile index (4,5), amount of inciso gingival show of the teeth (6–9), golden proportion (10,11), smile arc (7,10,12–14), and width of buccal corridors (5,10,15–17) on smile esthetics have been thoroughly evaluated, but there is currently no unanimity concerning the effects of special factors on producing a beautiful smile among these studies.

The question of whether the soft tissue of the lips and perioral or the teeth and gingiva visible in the smile have a more important role in smile esthetic perception was the basis of this study. The judgments of laypersons and restorative specialist groups were included to provide comparisons between orthodontists and other groups of judges concerning what constitutes a pleasing smile. Therefore, the aim of this study was to evaluate the effect of hard and soft tissues of the oral and perioral region on smile esthetic perception.

## Materials and Methods

Thirty women ranging between 20 and 30 years of age were enrolled in this case-control study. All subjects were normal occlusion females from the Mashhad Dental School with Class I molar and canine relationships and good anterior alignment who volunteered to be photographed while smiling. The subjects had no history of orthodontic treatment, no significant skeletal asymmetry, no anterior or posterior crossbite, and no missing or malformed anterior teeth. This study was approved by the University of Mashhad Ethics Committee, and all subjects provided their written informed consent. 

Photographs were taken using a digital camera (Panasonic Z30) with a Lumix lens. The distance between the photographic equipment and the subjects was 150 cm.

**Fig 1:**
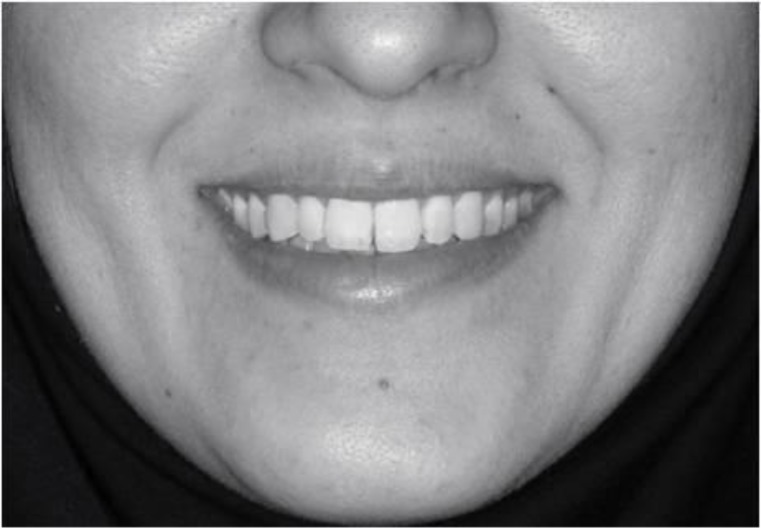
Full smile photograph

**Fig 2:**
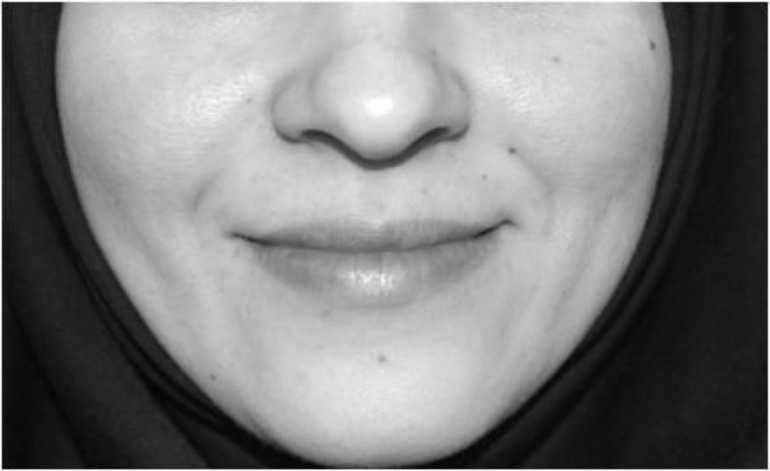
Lip-together smile photograph

**Fig 3:**
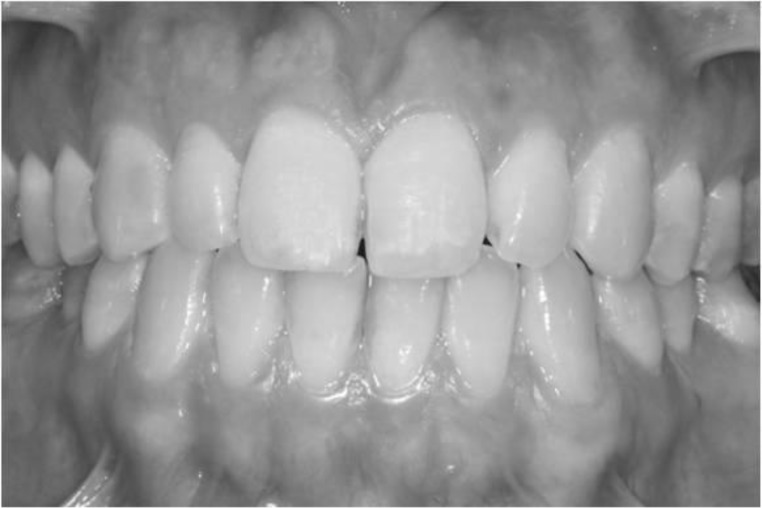
Frontal view of dentition photograph

After the subject's head was oriented into the natural head position, 5 photos were taken of each subject while smiling choose allow an unforced, natural smile to be selected. In addition, we took one photograph from dental frontal view of each subject using a retractor, as well as a lip-together smile photograph (Figs: 1,2,3). The subjects wore a head scarf in order to help the judges focus on the face rather than other extraneous features such as hair and neck shape. Thus, 30 images of the full smile, 30 of the lip-together smile, and 30 of the frontal view of dentition were taken.

 After downloading directly from the digital camera to the computer, the digital images were changed to black and white and converted into slides using PowerPoint software for projection onto a large screen. 

The evaluating panel consisted of 20 orthodontists, 20 restorative specialists, and 20 laypersons. Each group consisted of 10 men and 10 women ranging in age from 28 to 50 years. The male: female ratio was maintained at 1:1 in order to eliminate a gender bias. The raters were told that they would see 90 slides. They were asked to rate the attractiveness of the smiles on a 100-point scale, in which 0 demonstrated the ugliest and 100 the most beautiful form. The raters were shown each of the 90 slides in a random order for 15 s. Each rater made his or her evaluation privately, having no information about the subjects. Panel members were asked to re-evaluate the entire sample 5 weeks later.

The second evaluation by the panel members was found to be in the range of good repeatability (P= 0.83). An analysis of variance (ANOVA) and the Pearson correlation test were used for data analysis. In all statistical analyses, a P-value of less than 0.05 was considered statistically significant.

## Results

As shown in Table 1, the mean scores of the raters relating to teeth, lips, and full smiles were 53.12 ± 10.68, 51.87 ± 14.70, and 52.74 ± 13.07, respectively. The ANOVA revealed that there were no significant differences between mean scores of lips, teeth, and full smiles (P=0.930).

**Table 1:** Mean esthetic scores made by all judges (N=60) for the Teeth, Lips and Full Smile

	**Teeth**	**Lips**	**Full Smile**
Mean (±SD)	53.12 ± 10.68	51.86 ± 14.70	52.74 ± 13.07

Differences between the scores P-value (ANOVA) = 0.930

Table. 2 shows the individual mean scores of lips, teeth, and full smiles for orthodontists, restorative specialists, and laypersons. The ANOVA revealed there were no significant differences among the three groups in evaluating teeth (P=0.135) and lips (P= 0.466); however, the difference between raters for full smiles showed a significant difference (P=0.018). The scores of the orthodontist were lowest and those of the laypersons were highest.

**Table 2:** Mean esthetic scores made by Orthodontists (N=20), Restorative Specialists (N=20) and Laypersons (N=20) for the Teeth, Lips and Full Smile

	**Mean Teeth Score** **(±SD)**	**Mean Lips Score** **(±SD)**	**Mean Full Smile Score (±SD)**
Orthodontists Restorative	47.73 ± 9.86	47.44 ± 10.95	44.44 ± 10.95
Specialists	54.80 ± 10.89	55.66 ± 16.83	53.41 ± 16.63
Laypersons	56.83 ± 10.07	52.53 ± 15.75	60.37 ± 3.50
P-value (ANOVA)*	0.135	0.466	0.018

*Differences between the scores of the 3 groups of judges

Table. 3 shows that the correlation between the scores given by orthodontists to the full smile and each of its components was significant (α=0.05), and the amount of correlation of each component with the full smile was equal to 0.660 for teeth and lips. 

In addition, the table shows the correlation between the scores given to the full smile and each of its components (teeth and lips) in restorative specialists (α = 0.01, r =0.847, and r= 0.860 respectively). For laypersons there was no statistically significant correlation between the scores given to the full smiles and its components. Considering all raters, the correlation between the scores given to the full smile and teeth (0.680) was higher than the correlation between the full smile and lips (0.606).

**Table 3:** Correlation between the esthetic scores for the Full Smile and the Teeth and Lips.( Full Smile

	**Orthodontists** **(N=20)**	**Restorative Specialists (N=20)**	**Laypersons** **(N=20)**	**All judges** **(N=60)**
Teeth	0.660*	0.847**	-.0.030	0.680**
Lips	0.660*	0.860**	0.066	0.606**

Pearson Correlation Test** Correlation significant at the 0.01 level (2-tailed)*Correlation significant at the 0.05 level (2-tailed)

## Discussion

As harmony and balance are not fixed concepts, standards of beauty can vary among people with different ethnicity (9).This study was designed to define the effect of smile components on the perception of smile esthetics among 20 to 30 year-old Iranian females, who had a normal occlusion. The judges’ panel consisted of 20 orthodontists, 20 restorative specialists, and 20 laypersons. This study, contrary to other similar ones, did not investigate the details of a smile, such as buccal corridors (5,10, 15–17), smile arc (7,10,12–14), smile line (6–9), the shape of the teeth (21,23), golden proportion (10,11), and their effect on the perception of smile esthetics. Instead each smile was divided into two parts; the lips and the teeth (and gingiva); and the impact of each part was assessed on the total perception of smile esthetics.The result of this study showed that the highest mean score given by the raters was for teeth (53.12 ± 10.68), while the lowest was for lips (51.86 ± 14.70). There was no significant difference between them the highest and lowest scores. This could be explained by the fact that in this study, judges assessed black and white photographs. Elimination of beauty factors such as skin, teeth, and gingiva color could affect the judges' ratings, possibly making it difficult for them to accurately rate black and white photographs. For this reason, all the photographs were scored at approximately 50% based on the visual analog scale (VAS). It is possible that most of the judges needed access to a greater number of beauty factors in order to rate a profile as excellent.

In addition, this investigation showed in that contrast to a full smile, there was good agreement across the orthodontists, restorative specialists, and laypersons concerning teeth and lips. This study showed that laypersons scored smile the highest and orthodontists the lowest. In previous studies, Johnson and Smith and Kokich et al. also found that dental professionals were more sensitive to minor dental defects than laypersons (15,22). Among the three groups, restorative specialists scored a higher correlation between the components of smile esthetics and the beauty of the total smile. Even though the difference of the correlations of components and full smile was very small (0.847 vs. 0.860), this may be because restorative specialists have to pay close attention to detail in their small delicate operative field of work. This therefore makes them better at appraising the components of a smile and evaluating the effect of each one separately. The orthodontists group rated the second highest degree of correlation. In this group the correlation coefficient between the components of a smile and the full smile was also significant but lower than in the previous group. This can be explained by the fact that although the orthodontists appraised the components of the smile, their attention to detail was lower than for the restorative specialists because of their different working field. Since orthodontists work in the broader orofacial field, they deal with the teeth within the totality of the face. They therefore pay less attention to the fine details important to operative dentists. The level of education of the participants in the layperson group ranged from a high school diploma to an MS, and no one had a degree related to medical sciences. Lack of correlation between the perception of the beauty of the components and the full smile in this group demonstrates that laypersons do not observe the details of smiles, with the most important issue for them being the full smile. Furthermore, the separate pictures of the teeth and lips were meaningless to them, in contrast to the other groups, and they could not establish a connection between those pictures and the full smile of the same person.

For all the raters, the correlation between the scores given to the full smile and teeth (0.680) was higher than the correlation between the full smile and lips (0.606).

Although the difference was small, this could explain that for all raters, irrespective of their education and specialization, the teeth may be more important than the lips in the esthetic perception of full smile.

Until now, no similar study focusing on separating the components of the smile has been reported. Previous studies mostly considered the details of the smile, such as the buccal corridors (5,10,15–17), smile arc (7,10,12–14), smile line (6–8), and shape of the teeth (21,23), for example, on the total perception of the smile. Unlike this study, the studies which used different judging groups to evaluate the smile showed no difference among the groups, including the study reported by McNamara et al., which was performed to find the effects of hard tissue (cephalometric) and soft-tissue elements on smile esthetics. In that study there was also a panel of judges consisting of laypersons and orthodontists, and they concluded that the attitudes of the orthodontists and laypersons were consistent (r=0.93) (24). In another research accomplished by Schabel et al. (19) there was no statistically significant difference between the attitudes of the orthodontists and the patients’ parents toward smile esthetics. An interesting point in that study was that no objective measurement was capable of predicting the beauty or lack of beauty of a smile. 

This study showed there was no statistically significant correlation between the scores given to the full smile and its components for the layperson group. Combining the scores for the three judging groups together, there was no significant correlation between the ratings of the tooth, lips, and the overall smile.

Providing an ideal treatment has always been a duty for all specialists, including orthodontists, but this is not feasible in some situations due to the anatomical, physiolo- gical, and economic conditions of the patients. As it can be seen from this study, laypersons exhibit less attention to detail than specialists. 

## Conclusions

The role of teeth and lips in the esthetics perception of smile was similar for orthodontists. Restorative specialists are more influenced by the lips than the teeth. In contrast, laypersons failed to perceive the esthetics details and component of the smile. By considering all the evaluation of all raters, the role of the teeth seems more important than that of the lips in making a beautiful smile.
